# Human Breast Progenitor Cell Numbers Are Regulated by WNT and TBX3

**DOI:** 10.1371/journal.pone.0111442

**Published:** 2014-10-28

**Authors:** Lisa M. Arendt, Jessica St. Laurent, Ania Wronski, Silvia Caballero, Stephen R. Lyle, Stephen P. Naber, Charlotte Kuperwasser

**Affiliations:** 1 Developmental, Molecular, and Chemical Biology Department, Sackler School of Graduate Biomedical Sciences, Tufts University School of Medicine, Boston, Massachusetts, United States of America; 2 Molecular Oncology Research Institute, Tufts Medical Center, Boston, Massachusetts, United States of America; 3 Department of Cancer Biology, University of Massachusetts Medical School, Worcester, Massachusetts, United States of America; 4 Department of Pathology, Tufts Medical Center, Boston, Massachusetts, United States of America; Baylor College of Medicine, United States of America

## Abstract

**Background:**

Although human breast development is mediated by hormonal and non-hormonal means, the mechanisms that regulate breast progenitor cell activity remain to be clarified. This limited understanding of breast progenitor cells has been due in part to the lack of appropriate model systems to detect and characterize their properties.

**Methods:**

To examine the effects of WNT signaling and TBX3 expression on progenitor activity in the breast, primary human mammary epithelial cells (MEC) were isolated from reduction mammoplasty tissues and transduced with lentivirus to overexpress WNT1 or TBX3 or reduce expression of their cognate receptors using shRNA. Changes in progenitor activity were quantified using characterized assays. We identified WNT family members expressed by cell populations within the epithelium and assessed alterations in expression of WNT family ligands by MECs in response to TBX3 overexpression and treatment with estrogen and progesterone.

**Results:**

Growth of MECs on collagen gels resulted in the formation of distinct luminal acinar and basal ductal colonies. Overexpression of TBX3 in MECs resulted in increased ductal colonies, while shTBX3 expression diminished both colony types. Increased WNT1 expression led to enhanced acinar colony formation, shLRP6 decreased both types of colonies. Estrogen stimulated the formation of acinar colonies in control MEC, but not shLRP6 MEC. Formation of ductal colonies was enhanced in response to progesterone. However, while shLRP6 decreased MEC responsiveness to progesterone, shTBX3 expression did not alter this response.

**Conclusions:**

We identified two phenotypically distinguishable lineage-committed progenitor cells that contribute to different structural elements and are regulated via hormonal and non-hormonal mechanisms. WNT signaling regulates both types of progenitor activity. Progesterone favors the expansion of ductal progenitor cells, while estrogen stimulates the expansion of acinar progenitor cells. Paracrine WNT signaling is stimulated by estrogen and progesterone, while autocrine WNT signaling is induced by the embryonic T-box transcription factor TBX3.

## Introduction

The regenerative capacity of the mammary gland is driven by a complex cellular hierarchy that switches from multipotent stem cells during embryogenesis to hormone sensitive stem/progenitor cells shortly after birth [1]–[5]. Rodent studies have shown that ductal elongation at puberty is mediated primarily through estrogen, while side branching and lobule-alveolar development is driven largely through the combined actions of progesterone and prolactin [6]–[9]. However, due to differences in breast structure and circulating hormones between humans and mice, the effects of estrogen and progesterone during ductal elongation, side branching, and lobule-alveolar development have not been fully delineated. Genetic deletion and tissue transplant studies have suggested that estrogen and progesterone act through paracrine signaling to induce the proliferation of neighboring cells in mice [10]–[12]. Although paracrine mediators for estrogen and progesterone signaling have been elucidated in the mouse mammary gland during puberty [13]–[16], the effectors for steroid receptor signaling in the adult human breast are only recently being identified [17].

Prior to puberty, non-hormonal mechanisms regulate mammary stem/progenitors in mice. TBX3 induction is one of the earliest events during mammary gland specification and formation [18]–[20]. Heterozygous mutations in TBX3 result in ulnar-mammary syndrome (UMS) in humans, which results in severe mammary gland hypoplasia [21], [22], accentuating its importance during this developmental stage. Although *Tbx3* is expressed in adult mammary tissues in mice [20], little is known about its function after birth. Since adult *Tbx3* heterozygous mice have reduced ductal branching in all 5 pairs of mammary glands [23], it is likely that *TBX3* is necessary for mammary stem/progenitor cell proliferation following embryonic development. Functionally, TBX3 has been characterized as a transcriptional repressor, exerting its functions through its T-box DNA binding domain [24]. In other contexts, *TBX3* is necessary for the maintenance of stem cell self-renewal, cell-fate determination and organogenesis (for review, [25]–[27]). Together this suggests that TBX3 may play a role in the regulation of stem cell activity within the normal mammary epithelium.

During embryonic mammary development, TBX3 is thought to induce WNT signaling to promote the expansion and self-renewal of mammary embryonic stem cells [20], and WNT signals may in turn maintain TBX3 gene expression [19]. Unlike TBX3, gene expression for the WNT family ligands has been examined following embryonic development, and WNT signaling is active during all phases of mouse mammary gland development, including puberty, pregnancy, lactation and involution (for review [8], [28]). The most characterized mechanism of WNT family signaling is through the canonical pathway that is initiated by binding of WNT ligands to Frizzled-Lrp receptor complexes at the cell surface. WNT signaling has been shown to promote the long-term expansion and self-renewal of stem cells over multiple stages of development through this characterized canonical pathway [4], [29]. While some WNT ligands have been identified as potential downstream targets of steroid receptor hormones in the murine mammary gland [30], the regulation and identification of other family members in the human breast has not been examined.

In this study, we sought to examine the mechanisms that regulate human breast progenitor cell activity. In doing so, we reveal a complex interplay between estrogen, progesterone, TBX3 and WNT signaling that underlies human breast morphogenesis.

## Materials and Methods

### Primary Tissue Isolation and Culture

All human breast tissues were obtained in compliance with the laws and institutional guidelines, as approved by the Tufts Medical Center Institutional Review Board and UMass Institutional Review Board at the University of Massachusetts Medical School. The approval number for human subject research is 00004517. Non-cancerous breast tissues were obtained from patients undergoing elective reduction mammoplasty. De-identified breast tissue was utilized for these studies, and for this reason informed consent was not required. Breast tissues were enzymatically digested as previously described [31], [32], and primary mammary epithelial cells were transplanted *in vivo* for normal outgrowth studies or infected with lentivirus and grown in culture. For normal outgrowth studies, epithelial organoids were dissociated to single cells and infected in suspension with lentiviruses encoding CSCG GFP empty vector. Lentiviral particles were generated and concentrated as described [2].

### Animals

The care of animals and all animal procedures were conducted in accordance with a protocol approved by the Tufts University Institutional Animal Care and Use Committee, and the approval number for animal research is A-3775-01. Colonies of NOD/SCID mice were maintained in house. Mice were given food and water *ad libitum*. To humanize mice, mammary epithelium was removed from the fourth mammary glands of 3 week-old NOD/SCID females, and RMF-EG cells were injected into the fat pad as described [31]. Two weeks post-humanization, GFP virus-infected cells (100,000 per gland) were co-mixed with primary breast stromal cells (2.5×10^5^ per gland) in a 1∶1 mixture of collagen and Matrigel (BD Biosciences) and injected into humanized fat pads. Mice were ovariectomized at the time of transplantation and implanted with 0.1 mg 17β-estradiol (E2), 10 mg progesterone (P4), 0.1 mg E2/10 mg P4 or placebo pellets (Innovative Research of America) to model serum levels of hormones present during the luteal phase of the menstrual cycle. Tissue was collected 8 weeks following surgery and imaged for GFP expression.

### Cell lines and tissue culture

RMF-EG cells were grown in DMEM (Invitrogen) supplemented with 10% calf serum [32]. MCF10A cells (ATCC) were grown in mammary epithelial cell basal medium (MEBM; Lonza) supplemented with bovine pituitary extract (52 µg/mL), hydrocortisone (0.5 µg/mL), human EGF (10 ng/mL), and insulin (5 µg/mL; Lonza) and 100 ng/mL cholera toxin. Primary epithelial cells isolated from de-identified reduction mammoplasty tissues were cultured in MEBM supplemented with bovine pituitary extract (52 µg/mL), hydrocortisone (0.5 µg/mL), human EGF (10 ng/mL), and insulin (5 µg/mL) for mammosphere and adherent colony growth. All cells were grown at 37°C and 5% CO_2_, and media was supplemented with 1% penicillin/streptomycin (Invitrogen).

### MCF10A stable cell lines

Bacterial glycerol stocks of MISSIONshRNA were obtained (Sigma), and plasmid DNA was isolated by maxiprep (Qiagen). The VSV-G-pseudotyped lentiviral vectors were generated by transient cotransfection of the above vectors with the VSV-G-expressing construct pCMV-VSV-G and the packaging construct pCMV DR8.2Dvpr, both generously provided by Inder Verma (Salk Institute), into 293T cells with the FuGENE 6 transfection reagent (Roche). Viral supernatant was collected and introduced to subconfluent MCF10A cultures. Lentiviral integration was selected with 1 µg/mL puromycin (for shRNAs), 10 µg/mL blasticidin (TBX3, Empty Vector), or by FACS sorting for GFP (WNT1, Empty Vector) expression at Tufts University Pathology Core using Legacy MoFlo (Beckman Dickson).

### Colony forming assays

Dissociated primary mammary epithelial cells (MEC) were passed through a 20 µm filter (Millipore). Single MEC or MCF10A cells were plated on 6-well non-adherent plates (Corning) at 40,000 cells/well (primary cells) or 10,000 cells/well (MCF10A) as described [33] for mammosphere formation or on 6-well adherent plates at 35,000 cells/well (MEC) and 10,000 cells/well (MCF10A) for adherent and floating colony formation. Colonies were grown for 7 days. Floating colonies and mammospheres were quantified using a Multisizer3 Coulter Counter (Beckman Coulter). Mammospheres were collected by centrifugation (800 rpm) after 7 days and dissociated enzymatically with 0.25% trypsin (Invitrogen) and mechanically by triteration through a pipette. The cells obtained by dissociation were filtered though a 20 µm filter and plated at 20,000 cells/well on 6-well non-adherent plates. Secondary mammospheres were quantified after 7 days.

For the *in vitro* experiments, cells were plated with 1 ng/mL E2, 1 ng/mL P4, E2+P4, or vehicle in basal media. The choice of using 1 nM P4 for these studies was based on careful consideration of the concentrations of P4 used in prior studies [34]–[36], and preliminary experiments using various concentrations of P4, where we did not observe statistically different changes in the number or character of colonies formed in response to increasing doses of P4. In addition, the concentration of P4 and E2 used were chosen to model the dynamic physiological levels of these hormones during the menstrual cycle. Over the course of the menstrual cycle, the concentrations of E2 and P4 differ in relation to each other, and they are never found in the absence of one another. Even during the follicular phase when serum estrogen levels are significantly elevated compared to P4, P4 is not absent. All assays were run in triplicate.

Adherent colonies from primary cells were fixed in methanol and stained for cytokeratin 8 (1∶500, Vector) and 14 (1∶500, Thermo Scientific) as described [2]. MCF10A colonies were fixed in methanol and stained with crystal violet. Stain was extracted with 10% acetic acid and absorbance quantified at 540 nm. All assays were run in triplicate.

For collagen assays, 5,000 single primary cells or 1,000 MCF10A cells were plated on 1 mg/mL collagen and overlayed with 2% Matrigel in basal mammary media (Lonza) on 4 well chamber slides as described [2]. Mammospheres and floating colonies were quantified prior to plating on collagen. Collagen assays were performed in duplicate and quantified as described [2]. Briefly, colonies with one or more branches were determined to be ductal colonies, round-shaped colonies were defined as acinar colonies, and cells that spread as a monolayer on the collagen surface were determined to be flat colonies. All ductal and flat colonies that formed on the collagen surface were counted, as well as all acinar colonies that were greater than 50 µm in diameter. For hormone treatments, cells were plated with 1 ng/mL E2, 1 ng/mL P4, E2+P4, or vehicle in basal mammary media. For conditioned media experiments, TBX3 overexpressing and empty vector (EV) control cells were grown to confluence and conditioned media was collected after 24 hours. shscrambled control or shLRP6 cells were plated in conditioned media with 2% matrigel on 1 mg/mL collagen gels. Assays were plated in duplicate for 3 experiments and colonies were quantified after 7 days.

### Sorting epithelial progenitor cell populations

Digested cells were plated briefly in serum (1–2 h) to deplete mammary fibroblasts from the organoid fraction. The organoids remaining in suspension were dissociated by trypsinization and filtered with a 40 µm filter (BD Biosciences) to remove residual clustered cells. Single-cell suspensions of breast epithelial cells were sorted with CELLection pan-mouse IgG magnetic beads (Dynal; Invitrogen) conjugated to an anti-CD10 antibody (clone SS2/36; Santa Cruz Biotechnology) according to the manufacturer’s instructions. CD10^+^ cells were released from the beads by DNase treatment according to the manufacturer’s instructions and by incubation with occasional agitation at 37°C for 10 min; additional DNase I (5 µg/mL; Roche) was added to facilitate bead release if necessary. Cells that did not bind to the CD10 beads were further sorted with magnetic beads conjugated to an anti-EpCAM antibody (clone VU-ID9; Abcam and AbD Serotec). Positive cells were again released by DNase treatment. EpCAM^+^ bead-sorted cells were further sorted by binding of CD49f antibody (clone 450-30A; AbD Serotec) conjugated beads. Beads were released from positively sorted cells as described above. Viable cells (verified by trypan blue exclusion) from fractions enriched with unsorted, basal progenitor cells (BPC, EpCAM^−^CD10^+^), mature basal (MB; EpCAM^+/l^ °CD10^+^), luminal progenitor cells (LPC, EpCAM^+^CD49f^+^) and mature luminal (EpCAM^+^CD49f^−^) cells were used for collagen assays and quantitative real-time PCR (qPCR).

### Cell proliferation assay

Primary mammary epithelial cells were plated at 35,000 cells/well on adherent plates for 7 days. Epithelial cells were incubated with 50 µΜ 5-bromo-deoxyuridine (BrdU) for 1 hour prior to trypsinization. Cells were counted, permeabilized with 70% EtOH at 4°C for 1 hour, incubated with 2N HCL/0.5% Triton X-100 for 30 minutes, washed with 1% BSA in PBS, and incubated with 0.1 M Sodium Tetraborate. Epithelial cells were incubated with FITC-conjugated mouse anti-BrdU monoclonal antibody (clone MOPC21; BD Pharmingen) or isotype control (clone 3D4; BP Pharmingen) for 30 minutes at 4°C in 0.5% Tween-20/1% BSA in PBS. Cells were washed, resuspended in 0.5 mL PBS containing 10 µg/mL RNaseA and 20 µg/mL propidium iodide, and incubated in the dark at room temperature for 30 minutes. All samples were run on a FACSCalibur flow cytometer (BD Biosciences). Flow cytometry data were analyzed with the FlowJo software package (TreeStar).

### Quantitative PCR analyses

RNA was isolated utilizing RNeasy mini kit with on-column DNase digestion (Qiagen). RNA samples were reverse transcribed using iScript cDNA kit (Bio-Rad), and qPCR was performed with SYBR Green (Bio-Rad) on a CFX96 Real-Time System (Bio-Rad). Data was analyzed as a fold change utilizing ΔΔCt method or by relative expression with ΔCt. Samples were run in duplicate, and three experiments were analyzed. Primer sequences are listed in Table S1.

### Immunohistochemistry and immunofluorescence

Immunohistochemistry was performed on methanol-fixed adherent colonies as described [2], [37]. For immunofluorescence (IF), single primary epithelial cells were cultured for 7 days as mammospheres, then concentrated and spun onto slides using Cytospin4 (Thermo Scientific). IF staining for estrogen receptor alpha (ERα; 1∶100, Abcam) or progesterone receptor (PR; 1∶500, Cell Signaling Technologies) was performed as described [38]. Double labeling with CK8 (1∶1000, Vector) and PR or p63 (1∶500, Santa Cruz Technologies) and PR was performed on paraffin embedded reduction mammoplasty tissues from 8 patients. Sections underwent antigen retrieval in 0.1 M citrate buffer (pH = 6.0), were blocked for 1 hour with 5% bovine serum albumin in PBS, and incubated with primary antibodies overnight. Double labeling with TBX3 (1∶100, Abcam) and CD45 (1∶50, BD Biosciences) was performed on paraffin embedded reduction mammoplasty tissues from 5 patients. Sections underwent antigen retrieval in 0.1 M tris buffer (pH = 9.0), were blocked for 1 hour with 5% fish gelatin (Sigma) in TBST, and incubated with primary antibodies overnight. Sections were incubated with Alexa fluor 546 goat anti-rabbit IgG or Alexa fluor 488 goat anti-mouse IgG (1∶250, Invitrogen) for 30 minutes at room temperature. Sections were mounted with Vectashield mounting media with dapi (Vector) and coverslipped. Images were captured with Spot imaging software system (Diagnostic Instruments, Inc.).

### Western blotting

MCF10A cells were lysed, and protein concentrations were measured as described [38]. Immunoblotting was performed as described [38], and membranes were incubated with TBX3 antibody (1∶5000, Abcam), LRP6 (1∶1000, Cell Signaling), or WNT1 (1∶1000, Invitrogen). The membranes were stripped and re-hybridized with β-actin antibody (1∶1000, Abcam). For detection of proteins in WNT1 CM, cells were grown for 48 hours prior to media collection. CM was centrifuged through 30 kDa column (Millipore). Protein levels were quantified in the retentate. For detection of LRP6 and LRP5 in sorted cell populations, primary human epithelial cells were sorted into CD10^+^ and EpCAM^+^ cellular populations as described above. Unsorted, CD10^+^, and EpCAM^+^ cells were lysed, and protein concentrations were measured as described [38]. Immunoblotting was performed as described [38], and membranes were cut at the 75 kDa molecular weight marker and incubated with LRP6 (1∶1000, Cell Signaling), LRP5 antibodies (1∶1000, Cell Signaling), or β-actin antibodies (1∶1000, Abcam).

### Statistical analyses

Results for colony forming assays performed in triplicate or duplicate were expressed as the mean±s.e.m. Results from qPCR studies were expressed as mean±s.d. Statistical tests included unpaired two-tailed Student’s t-test (for 2 groups) or one-way repeated measures ANOVA, followed by multiple comparisons (for more than 2 groups). P values of 0.05 or less were considered to denote significance. Statistical analyses were performed using Graph Pad Prism (Graph Pad Software).

## Results

### TBX3 enhances progenitor activity in adult human breast epithelial cells

**Figure 1:**
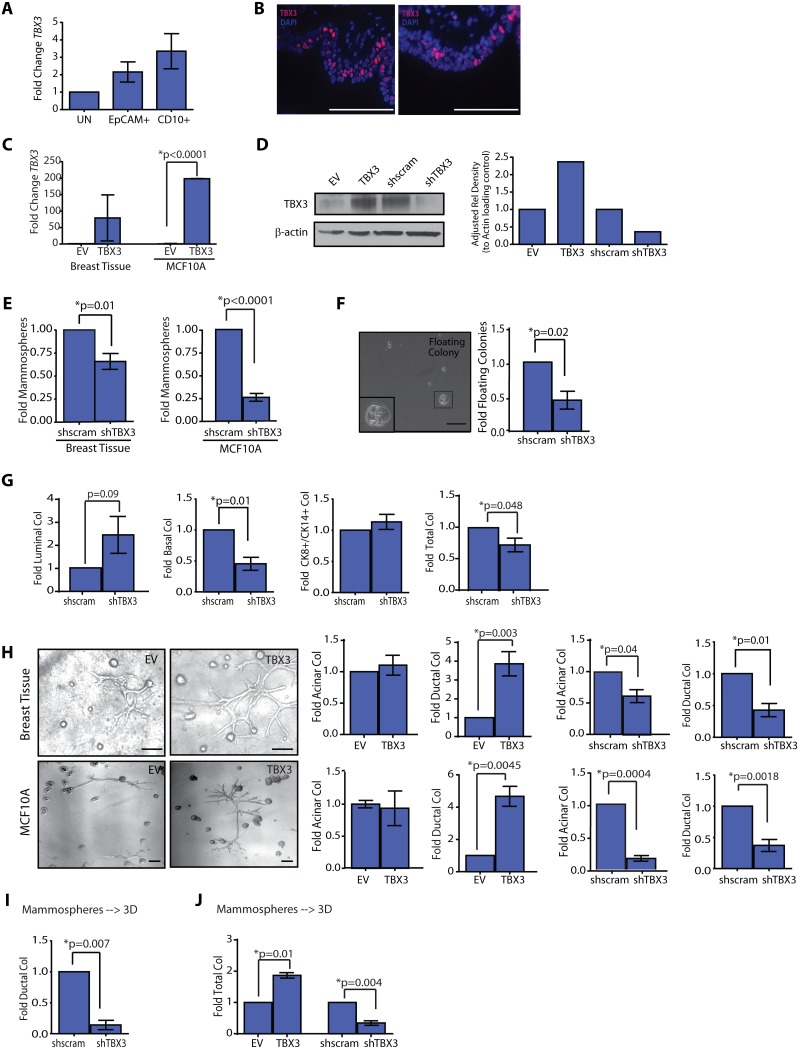
TBX3 overexpression enhances ductal progenitor activity. (**A**) TBX3 transcripts were detected in using qRT-PCR in unsorted (UN), luminal (EpCAM^+^) and basal (CD10^+^) primary mammary epithelial cells (MEC) isolated and sorted based on cell surface markers from reduction mammoplasty tissues (n = 3 patient samples). (**B**) Endogenous TBX3 was detected using immunofluorescence in both the luminal and basal layers of formalin-fixed, paraffin embedded human breast tissues. (**C, D**) MECs (n = 3 patient samples) and MCF10A cells (n = 3 experiments) were transduced with lentivirus for TBX3. TBX3 expression (mean±s.d.) was detected by qPCR (**C**) and Western blotting of cell lysates (**D**). MCF10A cells transduced with short hairpins for TBX3 demonstrated reduced TBX3 protein compared with shscrambled (shscram) control cells (**D**). (**E**) Reduction in TBX3 expression reduced mammosphere formation compared to shscram control cells in primary MEC (n = 3 patient samples; mean±s.e.m) and MCF10A cells (n = 3 experiments). (**F**) shTBX3 MECs significantly reduced floating colony formation over adherent cultures (n = 3 patient samples; mean±s.e.m). (**G**) Primary MEC infected with shTBX3 lentivirus formed significantly reduced numbers of basal (CK14^+^) and total colonies in adherent culture (n = 3 patient samples; mean±s.e.m). (**H**) TBX3 overexpression in MECs (n = 8 patient samples; mean±s.e.m) and MCF10A cells (n = 3 experiments) significantly increased ductal colony growth on collagen. shTBX3 MECs (n = 4 patient samples) and MCF10A cells (n = 3 experiments) demonstrated significantly fewer ductal and acinar colonies (mean±s.e.m.). (**I, J**) shTBX3 MECs demonstrated significantly reduced ductal (**I**) and total (**J**) colonies compared to shscram MECs, while TBX3 overexpression significantly increased total (**J**) colonies compared to EV MECs. MEC were grown as mammospheres prior to plating on collagen (n = 3 patient samples; mean±s.e.m.). Scale bars = 100 µm.

TBX3 expression is necessary for breast organogenesis and its aberrant expression has been identified in breast cancers, yet little is known about its function in epithelial cells of the adult human breast. To determine which cells in the breast expressed *TBX3*, primary epithelial cells isolated from reduction mammoplasty tissues were dissociated and sorted into luminal and basal epithelial cells, based on their cell surface expression of EpCAM and CD10, respectively [2], [37], [39], [40]. *TBX3* transcripts were detected using real-time quantitative PCR (qPCR) in both luminal and epithelial cell populations (Figure 1A). Consistent with this finding, TBX3 protein was detected using immunofluorescence in a discontinuous pattern in both the luminal and basal/myoepithelial layer of the breast epithelium (Figure 1B). Although CD45^+^ immune cells frequently invade through mammary epithelium and express TBX3, only a small percentage of TBX3^+^ cells also expressed CD45 (Figure S1A).

To delineate the effects of TBX3 on progenitor activity in mammary epithelial cells, we utilized both primary mammary epithelial cells (MEC) isolated from reduction mammoplasty tissues and MCF10A cells, an immortalized normal human breast epithelial cell line [41]. Primary MECs and MCF10A cells were transduced with full length human *TBX3* or with short hairpins targeting TBX3 (*shTBX3*). MECs and MCF10A cells overexpressing TBX3 exhibited a significant increase in expression (Figure 1C, D), while those transduced with shRNA targeting *TBX3* demonstrated a significant reduction in TBX3 expression compared to their respective controls (EV or shscram; Figure 1D).

To quantify the effects of TBX3 on progenitor activity, we utilized characterized *in vitro* assays, including growth in limiting dilution under non-adherent conditions, termed mammospheres, and adherent conditions. When MECs are grown under adherent culture conditions, lineage-restricted progenitors can be identified using immunohistochemistry to stain the adherent colonies: luminal progenitors which form cytokeratin (CK) 8^+^ colonies, mixed-lineage adherent colonies which express both CK8 and 14, and basal/myoepithelial progenitors, which form basal CK14^+^ colonies (Figure S1B; [2], [37], [42]). In addition, cells from the luminal EpCAM^+^ cell fraction have been shown to form restricted CK8^+^ colonies that float in suspension over the surface of adherent plates [2], [37] and can be extracted from the media and characterized. These diverse colony types are not observed in the MCF10A cell line when grown on adherent plates. TBX3 overexpression did not increase mammosphere or floating colony formation in primary epithelial cells or mammosphere formation in MCF10A cells (Figure S1C, D). However, shTBX3 MEC and MCF10A cells demonstrated a significant reduction in mammospheres (Figure 1E), and shTBX3 MEC demonstrated a significant reduction in floating colonies (p = 0.02; Figure 1F), compared with shscram control cells. Similarly, TBX3 overexpression did not alter colony formation (Figure S1E) on adherent plates, but reduction in TBX3 expression resulted in a significant decrease in basal (p = 0.01) and total (p = 0.048) colonies compared with shscram cells (Figure 1G), as well as significantly decreased total colony formation in shTBX3 MCF10A cells compared with shscram cells (p = 0.01; Figure S1F). These findings suggest that TBX3 is an important regulator of progenitor activity in the mammary epithelium.

MECs form distinct acinar, ductal, and flat colonies when grown on 3D collagen gels (Figure S1G). Acinar and ductal colonies grow as a single layer of mixed lineages of cells surrounding a hollow lumen, while flat colonies grow in a monolayer on the collagen (Figure S1G, H). Interestingly, MECs grown as mammospheres prior to plating on collagen were enriched ∼120-fold in acinar and ductal structural colonies, while MECs grown as floating colonies were significantly enriched ∼150-fold for acinar colonies (Table S2), suggesting that growth of MEC in suspension prior to plating on collagen enriched for specific types of colonies. TBX3, EV, shTBX3 and shscram MECs and MCF10A cells were grown in collagen to assess the functional role of TBX3 in luminal acinar and basal ductal colony formation. TBX3 overexpression significantly increased ductal colony formation in both MECs and MCF10A cells (Figure 1H) but had no effect on flat colony formation (Figure S1I). Conversely, inhibition of TBX3 expression attenuated both acinar as well as ductal colony formation (Figure 1H). This was observed in both primary MECs as well as in MECs enriched in progenitor cells by first growing them as spheres prior to plating on collagen (Figure 1I, J). These findings suggest that TBX3 regulates both acinar and ductal progenitor activity in the mammary epithelium.

### WNT signaling increases luminal acinar progenitor cell activity

During embryonic development, TBX3 and WNT family signaling are intrinsically linked [19], and both WNT and TBX3 are needed for mammary bud formation and primitive ductal elongation. Given the well-known role of WNT proteins in regulating mammary stem/progenitor cells [29], we examined whether WNT signaling might also affect progenitor activity and the relationship between WNT and TBX3 in human breast tissues. To assess WNT ligand expression within the human breast epithelium, we sorted primary MEC into luminal (EpCAM^+^) and basal (CD10^+^) cell populations and examined the expression of transcripts from the sorted cell population using qPCR. Multiple WNT ligands were expressed by both luminal and basal epithelial cells (Figure S2A). Given the diversity of WNT ligands expressed by human breast epithelial cells, we utilized WNT1, which signals through the canonical pathway and has been well-characterized in the mouse mammary gland (for review, [43]).

**Figure 2:**
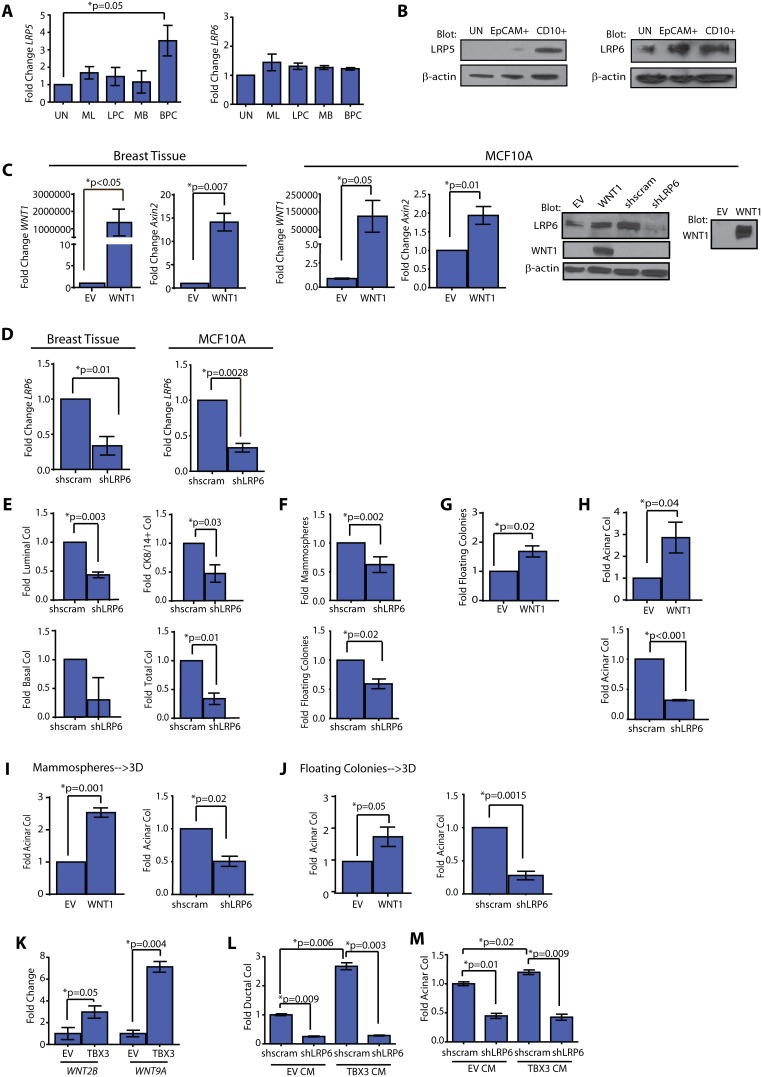
WNT enhances acinar progenitor activity through LRP6 signaling. (**A**) *LRP5* expression was enriched in basal progenitor cells (BPC; EpCAM^−^CD10^+^), while *LRP6* expression was present at similar levels in all fractions. Mammary epithelial cells (MECs) were sorted and expression levels were quantified by qPCR (n = 6 patient samples; mean±s.d.). UN = unsorted, ML = EpCAM^+^CD49f^−^, LPC = EpCAM^+^CD49f^+^, MB = EpCAM^+^CD10^+^. (**B**) LRP6 protein was detected in cell lysates from EpCAM^+^ luminal cells and CD10^+^ basal cells, while LRP5 protein was detected in lysates from CD10^+^ basal cells. (**C**) WNT1 overexpressing MECs (n = 3 patient samples) and MCF10A cells (n = 3 experiments) demonstrated significantly elevated expression of *WNT1* and downstream target, *axin2*. Expression was analyzed using qPCR (mean±s.d.). WNT1 protein was detected in cell lysates and conditioned media from WNT1 MCF10A cells. Protein for LRP6 was decreased in shLRP6 MCF10A cells compared with shscrambled (shscram) cells. Representative images from 3 experiments. (**D**) LRP6 expression was significantly reduced in MECs (n = 3 patient samples) and MCF10A cells (n = 3 experiments) transduced with shLRP6 lentivirus compared with those transduced with shscram. Expression was analyzed using qPCR (mean±s.d). (**E**) shLRP6 MECs demonstrated significantly reduced colony formation on adherent plates compared with shscram cells (n = 3 patient samples; mean±s.e.m). (**F**) shLRP6 MECs demonstrated reduced mammospheres and floating colony formation compared with controls (n = 4 patient samples; mean±s.e.m). (**G**) WNT1 MECs demonstrated significantly increased floating colony formation (n = 6 patient samples; mean±s.e.m.). (**H**) WNT1 MECs significantly increased acinar colony formation, while shLRP6 MECs significantly decreased acinar colony formation (n = 6 patient samples; mean±s.e.m.). (**I, J**) WNT1 expression increased acinar colonies, while reduced LRP6 expression diminished acinar colonies, when WNT1, EV, shscram, and shLRP6 expressing MECs were grown as mammospheres (**I**) or floating colonies (**J**) prior to plating on collagen (n = 5 patient samples; mean±s.e.m.). (**K**) TBX3 MCF10A cells demonstrated significantly increased expression of *WNT2B* and *WNT9A* compared with EV cells. Expression levels were quantified using qPCR and denoted as fold change compared to control cells (n = 3 experiments; mean±s.d.). (**L**) Ductal colonies were significantly increased in shscram MCF10A cells exposed to conditioned media (CM) isolated from TBX3 MCF10A cells compared to those grown in CM from EV MCF10A cells. Exposure to TBX3 CM did not enhance ductal colonies in shLRP6 MCF10A cells (n = 3 experiments; mean±s.e.m.). (**M**) shscram MCF10A cells demonstrated significantly increased acinar colony formation when treated with CM from TBX3 MCF10A cells compared to CM from EV MCF10A cells. Exposure to TBX3 CM did not enhance acinar colonies in shLRP6 MCF10A cells (n = 3 experiments; mean±s.e.m.).

WNT signaling requires the presence of cell surface receptors LRP5 or LRP6 in conjunction with Frizzled receptors. To determine whether progenitor cell populations expressed LRP5 or LRP6, freshly isolated MECs were sorted based on well-characterized cell surface markers [2], [37], [39], [40]: mature luminal cells (ML, EpCAM^+^CD49f^−^), luminal progenitor cells (LPC, EpCAM^+^CD49f^+^), mature basal/myoepithelial cells (MB, EpCAM^+^CD10^+^), and basal progenitor cell (BPC, EpCAM^−^CD10^+^), and we examined breast progenitor cell expression of *LRP5* and *LRP6* by qPCR. While BPCs expressed elevated levels of *LRP5* compared to other cell populations (p = 0.05), *LRP6* was highly expressed in all populations (Figure 2A). Similarly, LRP5 protein was detected in the CD10^+^ basal cell fraction, while LRP6 protein was detected in the luminal EpCAM^+^ and basal CD10^+^ cell fractions (Figure 2B). In order to examine WNT signaling, we transduced MCF10A cells and freshly dissociated MECs from reduction mammoplasty tissues with lentivirus encoding *WNT1* or with a short hairpin targeting the WNT co-receptor LRP6 (*shLRP6*).

WNT1-transduced MCF10A and MECs exhibited significant overexpression of WNT1 (p<0.05) as well as the canonical WNT downstream target, axin2 (p = 0.007; Figure 2C); WNT1 protein was also detected in the conditioned media (CM; Figure 2C). In cells transduced with *shLRP6* hairpins, *LRP6* expression levels were reduced by 3-fold (Figure 2C, D), with no change on the expression of endogenous WNT ligands (Figure S2B). To assess the effect of WNT1 expression on progenitor activity, WNT1 expressing MECs and MCF10A cells were seeded at clonal density on adherent, non-adherent, and collagen coated plates. WNT1 expression did not affect the number of luminal or basal colonies either on adherent plates (Figure S2C) or non-adherent plates (Figure S2D, E). However, when we inhibited WNT signaling by knock down of LRP6, we observed significant reductions in total adherent colony formation of primary MEC (Figure 2E, Figure S2F), and MCF10A cells (Figure S2G). In addition, shLRP6 MECs formed significantly fewer mammospheres (p = 0.002) and floating colonies (p = 0.02; Figure 2F) suggesting WNT signaling is necessary for the maintenance of human progenitor cells.

Although WNT1 overexpression did not affect progenitor cell numbers under adherent and mammosphere conditions, it did have a significant effect on the number of floating luminal progenitor colonies. A 1.8-fold increase in floating colonies was observed upon WNT1 overexpressing cells compared to EV control cells in MECs (p = 0.02, Figure 2G). Consistent with this observation, WNT1 overexpression also significantly increased acinar colony formation in collagen (p = 0.04; Figure 2H, Figure S2H). Inhibition of WNT signaling by *shLRP6* knock down abolished acinar colony formation in both MECs and MCF10A cells (p<0.001; Figure 2H; p = 0.001; Figure S2H), suggesting WNT signaling is necessary for luminal acinar progenitor activity. A similar result was observed when cells were first enriched for progenitors by growth as mammospheres (Figure 2I) or floating colonies (Figure 2J) prior to plating in collagen. No effects were observed on flat colony formation in WNT overexpressing MEC (Figure S2I). Taken together, these findings reveal that WNT signaling plays an important role in luminal acinar progenitor function.

Given that both WNT and TBX3 alter progenitor cell activity, we examined whether the regulation of human progenitor cell activity by TBX3 was mediated through WNT signaling. To assess the effect of TBX3 overexpression on WNT ligands, transcript levels of all *WNT* family ligands were evaluated in MCF10A cells overexpressing TBX3. Of all the WNT ligands examined, there was a significant 3-fold increase in *WNT2B* expression, and 7-fold increase in *WNT9A* expression in TBX3 overexpressing cells compared to EV control cells (Figure 2K). To determine if WNT signaling might be mediating the effects of TBX3 overexpression, MCF10A cells were grown on collagen gels in the presence of CM harvested from TBX3 overexpressing MCF10A cells. Compared to CM from control cells, CM from TBX3 expressing cells caused a significant increase in ductal colony formation (p = 0.006; Figure 2L). Knockdown of LRP6 could completely attenuate ductal colony formation induced by CM from TBX3 expressing cells implying that WNT signaling is the major downstream mediator of TBX3 in human MECs.

Given its expression in both the luminal and basal epithelial layers, we speculated that WNT production by TBX3 might also affect luminal progenitor activity. To examine this, we collected CM from TBX3 overexpressing cells and assessed whether it might be able to promote luminal acinar colony formation. Indeed, compared to CM harvested from control cells, CM from TBX3 expressing cells exhibited significantly increased luminal acinar colony formation activity in shscram cells (p = 0.02; Figure 2M). In contrast, luminal acinar colony formation was not enhanced in shLRP6 cells treated with TBX3 CM (p = 0.009; Figure 2M). These results suggest that TBX3 expression can induce WNT expression, and signal in an autocrine and paracrine manner to promote progenitor activity.

### Differential hormone receptor expression and activity in human breast progenitor cells

**Figure 3:**
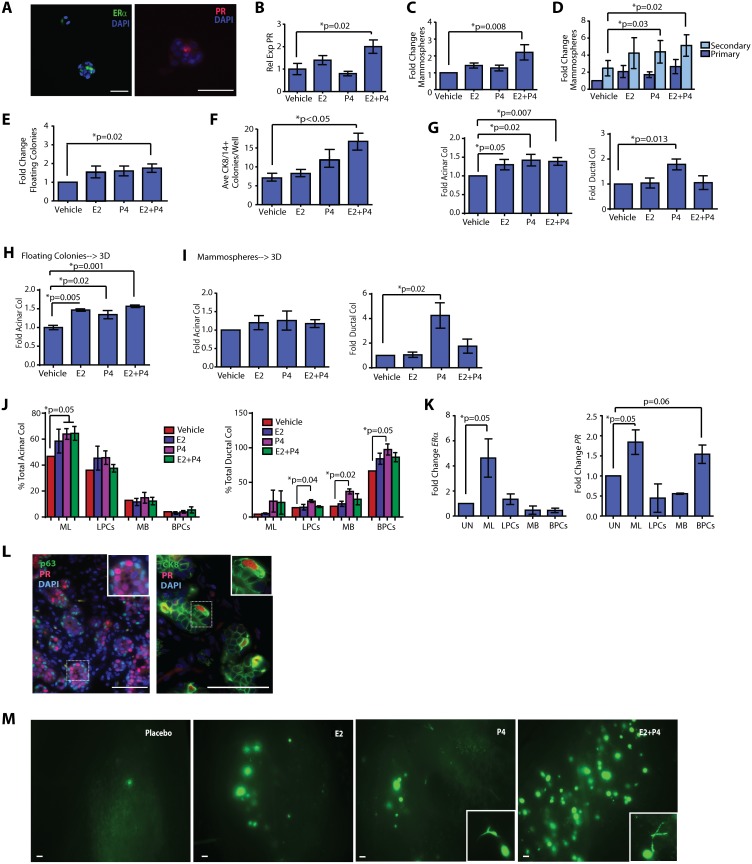
Progesterone increases basal ductal progenitor activity. (**A**) Mammospheres formed from mammary epithelial cells (MECs) expressed estrogen receptor alpha (ERα) and progesterone (PR) receptor. (**B**) Mammospheres treated with E2+P4 significantly upregulated PR expression. PR expression was detected by qPCR (n = 3 patient samples; mean±s.d.). (**C**) 17β-estradiol (E2) + progesterone (P4) treatment significantly enhanced MEC mammosphere numbers (n = 9 patient samples; mean±s.e.m.). (**D**) P4 and E2+P4 treatment significantly enhanced secondary mammosphere numbers (n = 6 patient samples; mean±s.e.m.). (**E**) E2+P4 treatment of MECs significantly increased floating colony growth (n = 9 patient samples; mean±s.e.m.). (**F**) E2+P4 treatment of MECs significantly enhanced the growth of bi-potent (CK8/14^+^) colonies in adherent culture (n = 3 patient samples; mean±s.e.m.). (**G**) On collagen, E2, P4, and E2+P4 significantly increased acinar colonies. P4 significantly enhanced ductal colonies (n = 9 patient samples; mean±s.e.m.). (**H**) E2, P4, and E2+P4 significantly increased acinar colonies. MECs were grown as floating colonies, then plated with hormones on collagen (n = 3 patient samples; mean±s.e.m.). (**I**) P4 significantly increased ductal colonies. MECs were grown as mammospheres, then plated on collagen with hormones (n = 7 patient samples; mean±s.e.m.). (**J**) P4 and E+P significantly increased acinar colonies in mature luminal (ML; EpCAM^+^CD49f^−^) cells, and P4 significantly increased ductal colonies in luminal progenitor cells (LPC; EpCAM^+^CD49f^+^), mature basal (MB; EpCAM^+^CD10^+^), and basal progenitor cells (BPC; EpCAM^−^CD10^+^). MECs were sorted and grown on collagen in the presence of hormones (n = 3 patient samples; mean±s.e.m.). Data are represented as a fold change compared to the vehicle control for each cellular population multiplied by the percentage of total acinar or ductal colonies formed. (**K**) ERα expression was enriched in ML cells; PR expression was enriched in ML and BPCs. MECs were sorted and qPCR performed (n = 6 patient samples; mean±s.d.). (**L**) PR expression was present in the basal epithelial layer adjacent to p63 expressing epithelial cells (inset). Basally located PR expressing cells also expressed CK8 (inset). (**M**) Primary epithelial cells (MEC) were isolated from reduction mammoplasty tissues, transduced with GFP lentivirus, and grown in the humanized fat pads of ovariectomized NOD/SCID mice treated with E2, P4, E2+P4, or placebo pellets. Glands from P4 and E2+P4 treated mice demonstrated increased growth of ductal structures (inset; n = 3 experiments). Scale bars = 100 µm.

Ovarian steroids are the master regulators of breast epithelial expansion during puberty and pregnancy. To determine how WNT and TBX3 signaling interact with ovarian hormones to regulate progenitor activity in MEC, we first delineated the effects of ovarian steroids on human breast progenitor cells. Although strides have been made in assessing the role of steroid hormones on progenitor activity [17], [44]–[47], the ability to examine the mechanisms of hormonal regulation in breast progenitor cells has been limited by a paucity of model systems that preserve functional signaling through hormone receptors. To establish an *in vitro* system to functionally dissect the effects of hormones on progenitor cell activity, MECs were plated at clonal densities under mammosphere conditions in the presence of 1 nM 17β-estradiol (E2), 1 nM progesterone (P4), 1 nM E2+P4, or vehicle for 7 days. Mammospheres contained cells that expressed estrogen receptor alpha (ERα) and progesterone receptor (PR; Figure 3A), and MEC treated with E2+P4 demonstrated a significant increase in *PR* expression compared with vehicle-treated MEC (p = 0.02; Figure 3B). Mammosphere formation was not affected in response to either E2 or P4 alone (Figure 3C). However, in response to E2+P4 treatment, the number of mammospheres formed was significantly increased (p = 0.008; Figure 3C), without a significant change in mammosphere size. Both P4 and E2+P4 treatment significantly enhanced secondary mammosphere formation (Figure 3D), suggesting that P4 and E2+P4 increased progenitor cell populations.

Treatment with E2 or P4 only modestly increased the number of floating colonies (Figure 3E), and alone did not affect the number of adherent colonies (Figure 3F, Figure S3A). However, combined E2+P4 treatment significantly increased the number of both floating colonies and mixed-lineage adherent colonies (Figure 3E, F). Although E2+P4 significantly enhanced colony formation, cellular proliferation was not significantly increased. E2+P4 treatment did not alter the number of cells in each phase of the cell cycle (Figure S3B) or the number of proliferating cells, as measured by BrdU incorporation (Figure S3C), in adherent culture. Together, these results suggest that the cooperation of both estrogen and progesterone can stimulate progenitor cell expansion.

To evaluate how these structural progenitors responded to E2 and P4, dissociated MECs were either plated on collagen as single cells or grown in suspension first to enrich for progenitors. Flat colonies were not increased in response to hormones (Figure S3D). Both single cells and sphere-grown MECs treated with E2, P4 or E2+P4 exhibited increased acinar colony formation on collagen (Figure 3G, H). Moreover, in the presence of P4 alone, single cells and sphere-grown MECs exhibited a significant increase in ductal colony formation (Figure 3G, I; p = 0.013; p = 0.02), suggesting that P4 regulates ductal progenitor activity.

To more specifically define which progenitor cell populations were responsive to hormones, we grew sorted MEC on collagen gels. Consistent with a prior report [2], when grown as 3D structures on collagen, ML/LPC cells were enriched in acinar colony formation, while MB/BPCs were enriched in ductal colony formation (Figure 3J). In response to hormones, however, an increase in acinar and ductal progenitor activity was observed only when P4 was present (Figure 3J).

Given these findings, we sorted MECs and examined the expression of the two major hormone receptors, *ERα* and *PR*. As expected, both receptors were robustly expressed in ML cells (Figure 3K). However, *PR* expression was also strongly expressed in cells enriched for BPCs (Figure 3K). Consistent with these findings, immunofluorescence studies conducted on intact normal breast tissue revealed PR expression in cells located within the luminal layer as well as basally located, adjacent to cells expressing the basal marker, p63 (mean = 11.5% PR expressing cells; Figure 3L). These data are in agreement with recent findings in which PR expression can be found in basal cells [46], [48]. Interestingly, these basally located PR cells also co-expressed CK8 (Figure 3L), which could potentially indicate the progenitor-like nature of these cells.

Finally, to examine the effects of hormones on human progenitor cells *in vivo*, MECs were directly dissociated from reduction mammoplasty tissues and injected into humanized glands of ovariectomized NOD/SCID mice supplemented with either 0.1 mg E2, 10 mg P4, 0.1+10 mg E2+P4, or placebo pellets. Compared to untreated mice, mice treated with hormones exhibited an increase in outgrowths; ductal outgrowths were primarily detected P4 or E2+P4-treated mice (Figure 3M, inset; Figure S3E), while acinar outgrowths were detected in all hormone-treated mice. Furthermore, compared to either E2 or P4 alone, the combination of E2+P4 significantly increased both the number and complexity of acinar and ductal outgrowths (Figure 3M; Figure S3E). These data indicate that hormones promote human mammary progenitor cell activity and further suggest differential regulation of acinar and ductal growth in response to hormones within the humanized glands.

Taken together, these findings suggest that both luminal and basal progenitor cells are sensitive to hormones. While the presence of both E2 and P4 together increases progenitor cell numbers, E2 favors acinar progenitor activity, and P4 favors both ductal and acinar progenitor activity.

### Autocrine and paracrine WNT signaling is necessary for acinar and ductal progenitor activity in response to hormones

**Figure 4:**
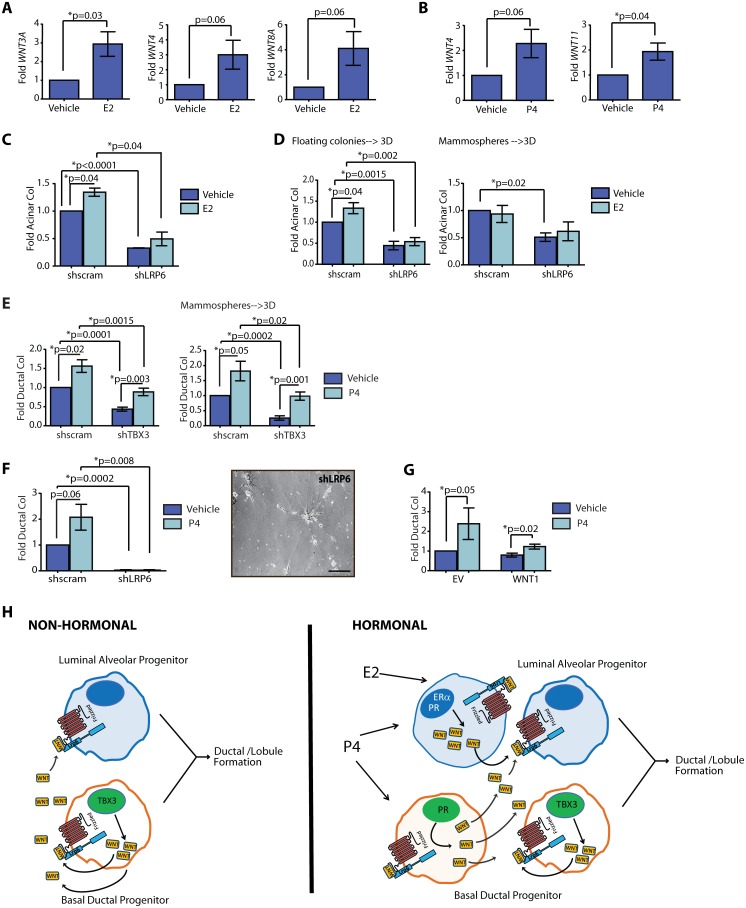
Breast progenitor activity is expanded through non-hormonal and hormonal mechanisms. (**A**) 17β-estradiol (E2) increased the expression of *WNT3A*, *WNT4*, and *WNT8A* in mammospheres compared to those treated with vehicle (n = 6 patient samples, mean±s.d.). Differences were detected by qPCR. (**B**) Progesterone (P4) increased the expression of *WNT4* and *WNT11* in mammospheres compared to those treated with vehicle (n = 6 patient samples, mean±s.d.). Differences were detected by qPCR. (**C, D**) shLRP6 mammary epithelial cells (MECs) demonstrated reduced acinar colony formation compared to shscram MECs when grown as single cells (**C**), or in suspension prior to plating on collagen (**D**), and E2 did not augment progenitor activity. E2 significantly increased acinar colony formation in shscram MECs compared to vehicle (n = 6 patient samples; mean±s.e.m.). (**E**) P4 increased ductal colony formation in shTBX3 MECs when grown as single cells or as mammospheres prior to plating on collagen (n = 6 patient samples; mean±s.e.m.). (**F**) shLRP6 MECs demonstrated decreased ductal colonies compared to shscram MECs on collagen. P4 did not enhance ductal colonies in shLRP6 MECs compared with vehicle (n = 6 patient samples; mean±s.e.m.). Reduction of LRP6 expression resulted in single cells with stellate projections instead of the growth of ductal colonies. (**G**) P4 treatment increased ductal colonies in WNT1 MECs compared with vehicle-treated cells (n = 6 patient samples; mean±s.e.m.). (**H**) TBX3 mediates non-hormonal growth through the secretion of WNT ligands. These ligands bind to LRP6 receptors on ductal progenitor cells as well as LRP6 receptors on acinar progenitor cells to coordinate the expansion of the two lineages for ductal/lobule formation. In response to hormones, cells expressing ERα and PR in luminal cells or PR in basal cells secrete WNT ligands to link the expansion of the two progenitor lineages for ductal/lobule formation in response to the cyclic changes of hormones during the menstrual cycle or pregnancy. Scale bar = 100 µm.

Our findings that E2 promotes acinar colony formation and P4 promotes ductal colony formation suggest that hormones may regulate progenitor cells through secreted WNT ligands and TBX3 activity. Consistent with this, during pregnancy in mice, paracrine WNT signaling is necessary for ductal/lobule and alveolar differentiation in response to hormones [8]. To assess whether E2 or P4 altered WNT ligand secretion, we grew primary MECs in non-adherent culture for 7 days and examined the effects of homones on WNT ligand transcripts. Both E2 and P4 increased expression of multiple WNT ligands, including *WNT3A*, *WNT4*, *WNT8A*, and *WNT11* (Figure 4A, B; Figure S4).

To determine whether the hormonal responsiveness of progenitors requires WNT signaling, LRP6 was inhibited in primary MECs dissociated from reduction mammoplasty tissues. shLRP6-MECs were grown on collagen in the presence of E2, P4, E2+P4 or vehicle and evaluated for progenitor activity. While E2 treatment stimulated acinar colony formation in control cells, knock down of LRP6 abolished this activity (Figure 4C). Similarly when MECs were grown as floating colonies or mammospheres prior to plating on collagen, *shLRP6* attenuated acinar colony formation (Figure 4D). This indicates that LRP6 is necessary for the E2-mediated increase in luminal acinar progenitor activity.

Since ductal progenitor activity was increased by P4, we also examined whether inhibition of WNT or TBX3 might be responsible for hormone responsiveness. shTBX3 MECs were grown on collagen either as single cells or as mammospheres prior to plating in the presence of P4. Despite TBX3 knock down, the effect of P4 on ductal progenitor activity was not altered, suggesting that TBX3 regulation of basal ductal progenitor activity occurs through a hormone independent mechanism (Figure 4E). Interestingly, *WNT9A*, which was increased by TBX3 overexpression (Figure 2K) was not upregulated in non-adherent culture by supplementation with hormones (Figure S4). In contrast, *shLRP6* not only abolished P4 induced ductal colony formation in collagen (p = 0.0002), the resulting structures were composed of single cells with stellate projections (Figure 4F). In addition, overexpression of WNT1 in MECs treated with P4 significantly increased basal ductal colony formation compared with vehicle-treated cells (p = 0.02; Figure 4G). Together, these results suggest that both acinar and ductal progenitor cells respond to hormones through WNT signaling.

## Discussion

The epithelium of the breast undergoes dramatic developmental changes, and cues for mammary epithelial morphogenesis shift from the localized diffusion of growth factors to systemic regulation by ovarian steroids and circulating hormones. Consistent with this, here we show that that mammary progenitor cells in human breast tissues are regulated by both hormonal and non-hormonal mechanisms (Figure 4H). However, in contrast to rodents where estrogen promotes ductal elongation and its inhibition can deplete stem cells [44], we found that estrogen directly enhances acinar progenitor activity in human cells through paracrine secretion of WNT ligands. In addition, unlike in mice where progesterone increases alveolar proliferation and expansion, progesterone stimulated human ductal progenitor cells and ductal morphogenesis through autocrine WNT signaling.

The species differences in hormonal regulation of progenitor cells may be due to the inherent differences in circulating levels and hormonal cycles that differ between species. During puberty in the mouse, estrogen is responsible for maturation of the mammary gland by mediating ductal elongation through mitogenic actions on stem/progenitor cells [11], [49]. Although estrogen is known to promote proliferation of human breast cancer cells [50]–[52], proliferation of normal cells within the human gland is not at its peak during the follicular phase, when circulating estrogens are at their maximum, but rather are maximal during the luteal phase, when the ratio of circulating progesterone to estrogen is increased [53], [54]. Uniquely, the human corpus luteum secretes estrogen in addition to progesterone [55], and tamoxifen administration can inhibit breast epithelial proliferation during the luteal phase of the menstrual cycle [56], suggesting that both estrogen and progesterone regulate progenitor activity in the human breast. This difference in estrogen to progesterone ratios during hormone cycles as well as differences in circulating estrogen levels may have important consequences in the regulation of stem/progenitor cells in higher mammals.

Despite the differences in the progenitor specific to hormonal responses, several similarities in progenitor cell regulation were observed between mice and humans. Combination estrogen and progesterone stimulated maximum stem/progenitor cell expansion, which has also been observed in rodents [44], [45]. Likewise, our studies reveal that WNT ligand receptor LRP6 is expressed by luminal epithelial cells and functionally contributes to the expansion of luminal acinar colonies. This pathway is a major regulator of mammary stem cells in mice [4], [29]. Proliferation and expansion of progenitor cells occurs through a RANKL-mediated pathway through luminal RANK expression [17]. Furthermore, our findings revealed that TBX3 coordinates a non-hormonal expansion of both acinar and ductal progenitor cells by inducing paracrine WNT signaling (Figure 4H). TBX3 regulation of WNT signaling has also been observed in mice [20], [57]. These parallels suggests that although the regulation of progenitor cells by ovarian steroids may differ in higher mammals, the localized growth factor signaling pathways that are ultimately responsible for their proliferation may be conserved between species.

WNT signaling has been shown to be important for stem cell regulation multiple epithelial tissues and in many organisms. The *WNT* family is complex, made up of multiple receptors including *Frizzled*, *LRP5,* and *LRP6*, as well as secreted ligands and inhibitors with limited ability to diffuse in the mammary matrix. Our data demonstrate that WNT signaling through LRP6 regulates both acinar and ductal colony formation. The specificity of WNT signaling to each progenitor type may be regulated by cell type specific expression of receptors including basal cell expression of LRP5, as well as secretion of inhibitors, DKK and SFRP. In the murine mammary gland, DKK3 expression is enriched in the basal epithelium suggesting that this may be a point of regulation [58].

We have also demonstrated that TBX3 upregulates *WNT9A* expression, a novel WNT protein not previously characterized for its role in the mammary gland or stem cell activity. Thus, the regulation and specificity of specific WNT ligands is likely to be important in WNT activity. Interestingly, mutations in *TBX3* have recently been identified in breast cancers [59] suggesting that misregulation of TBX3 may be important for the genesis and pathobiology of breast cancer. Indeed, estrogen has recently been reported to expand breast cancer stem-like cell cells through upregulation of TBX3 [52]. Furthermore, TBX3 activity is increased by WNT signaling in various cancer cell lines [60], [61]. These observations, suggest that estrogen signaling and *TBX3* mutations in the context of breast cancer may be important for deregulating stem/progenitor cell activity, a prerequisite for neoplastic transformation. Further studies will be necessary to characterize the role and biological significance of *TBX3* mutations in breast tumors and how they synergize with estrogen to drive cancer.

## Supporting Information

Figure S1 **TBX3 expression does not co-localize with CD45 expressing immune cells. (A)** TBX3 (red) was expressed by mammary epithelial cells as well as infiltrating CD45^+^ (green) immune cells in breast epithelium (arrows). Expression was detected by immunofluorescence on paraffin-embedded breast epithelial sections (n = 5 patient samples). Nuclei were counterstained with DAPI (blue). **(B)** Primary mammary epithelial cells (MEC) from reduction mammoplasty tissue plated at limiting dilution on adherent plates formed cytokeratin (CK) 8^+^ luminal colonies, CK14^+^ basal colonies, and mixed CK8/14^+^ colonies. **(C)** TBX3 overexpression in primary MEC (n = 3 patient samples; mean±s.e.m.) and MCF10A cells (n = 3 experiments) did not enhance mammosphere formation. **(D)** Overexpression of TBX3 in primary MEC did not enhance floating colony formation above adherent plates (n = 3 patient samples; mean±s.e.m.). **(E)** Overexpression of TBX3 in primary MEC did not enhance colony formation of any lineage on adherent plates (n = 3 patient samples; mean±s.e.m.). **(F)** Overexpression of TBX3 did not enhance total colony formation in MCF10A cells, however reduction in TBX3 expression resulted in significantly decreased total colony formation. Colonies were stained with crystal violet, and absorbance was quantified for 3 experiments in triplicate (mean±s.e.m.). **(G)** Growth of primary MEC and MCF10A cells on a collagen substrate results in the formation of 3 distinct colonies: luminal acinar, basal ductal, and flat colonies. **(H)** Acinar and ductal colonies growing on 3D collagen gels form a hollow lumen surrounded by a single layer of epithelial cells that demonstrate variable expression of CK8 and CK14. **(I)** TBX3 overexpression did not alter flat colony growth in MEC transduced with TBX3 lentivirus compared to MEC transduced with empty vector (EV; n = 8 patient samples; mean±s.e.m.). Scale bars = 100 µm.
(TIF)

Figure S2 **WNT1 expression increases luminal acinar progenitor cells.** **(A)** EpCAM^+^ luminal cells expressed significant levels of *WNT3* and *WNT4*, while CD10^+^ basal epithelial cells expressed increased levels of all *WNT* family ligands examined (n = 6 patient samples; mean±s.d.). Mammary epithelial cells (MECs) from reduction mammary samples were sorted and differences were detected using qPCR. **(B)** WNT family ligand expression was not significantly altered in MCF10A cells transduced with *shLRP6* compared with control cells (n = 3 experiments; mean±s.d.). Differences detected by qPCR. **(C)** WNT1 expression did not alter colony formation on adherent plates in MEC infected with WNT1 or empty vector (EV) lentivirus (n = 3 patient samples; mean±s.e.m.). **(D)** No difference in mammosphere formation was detected between control and WNT1 infected primary MECs plated at clonal density on non-adherent plates for 7 days. **(E)** No differences were detected for mammosphere formation in MCF10A cells expressing WNT1 compared with EV control cells or in shLRP6 cells compared with shscrambled (shscram) control cells (n = 3 experiments; mean±s.e.m.). **(F)** Diminished LRP6 expression significantly decreased colony formation on adherent plates in MECs infected with shLRP6 or shscram lentivirus (n = 3 patient samples). **(G)** Decreased expression of LRP6 significantly decreased colony formation in MCF10A cells. Colonies were stained with crystal violet, and absorbance was quantified for 3 experiments in triplicate (mean±s.e.m.). **(H)** WNT1 expression in MCF10A cells significantly increased acinar colonies compared with EV control cells. Decreased LRP6 expression significantly decreased both acinar colonies and ductal colonies compared with shscram control cells (n = 3 experiments; mean±s.e.m.). **(I)** WNT1 expression did not significantly alter flat colony formation compared to EV controls in lentivirally transduced MEC (n = 6 patient samples; mean±s.e.m.). Scale bars = 100 µm.
(TIF)

Figure S3 **Progesterone increases the formation of ductal outgrowths in humanized mammary fat pads, related to** **Figure 3** **. (A)** Treatment of mammary epithelial cells (MECs) with 17β-estradiol (E2) and/or progesterone (P4) did not increase the number of luminal or basal colonies in adherent culture compared to those treated with vehicle (n = 3 patient samples; mean±s.e.m.). **(B, C)** Growth of MEC with E2 and/or P4 in adherent culture did not alter the proliferation of colonies compared with those grown with vehicle (n = 3 patient samples; mean±s.e.m.). Proliferation was assessed by flow cytometry measuring cellular populations in each portion of the cell cycle when stained with propidium iodide **(B)** as well as by 5-bromo-deoxyuridine (BrdU) incorporation **(C)**. **(D)** MEC treated with E2 and/or P4 did not increase flat colony formation compared with those treated with vehicle (n = 9 patient samples; mean±s.e.m.). **(E)** Representative whole mounts and hematoxylin and eosin (H&E) stained sections from human-in-mouse (HIM) NOD/SCID mice. Primary epithelial cells (MEC) were isolated from reduction mammoplasty tissues, transduced with GFP lentivirus, and grown in the humanized fat pads of ovariectomized NOD/SCID mice treated with E2, P4, E2+P4, or placebo pellets. E2+P4 significantly enhanced the formation of acinar and ductal structures within the humanized glands. Glands from P4 and E2+P4 treated mice demonstrated increased growth of ductal structures (inset; n = 3 experiments). Scale bars = 100 µm.
(TIF)

Figure S4 **Expression of WNT family ligands in mammospheres is increased by estrogen and progesterone, related to** **Figure 4** **.** Expression of WNT family ligands was increased by treatment with 17β-estradiol (E2) and/or progesterone (P4) in primary epithelial cells grown as mammospheres compared with those treated with vehicle (n = 6 patient samples; mean±s.d.). Differences were detected by qPCR.
(TIF)

Table S1 **Primers for qPCR analysis.**
(DOCX)

Table S2 **Growth in suspension enhances structural progenitor activity.**
(DOCX)
