# Supplementary figures and images for: Human Breast Progenitor Cell Numbers Are Regulated by WNT and TBX3
Source: PLoS One. 2014 Oct 28;9(10):e111442. doi: 10.1371/journal.pone.0111442 (PMC4211891; doi:10.1371/journal.pone.0111442)

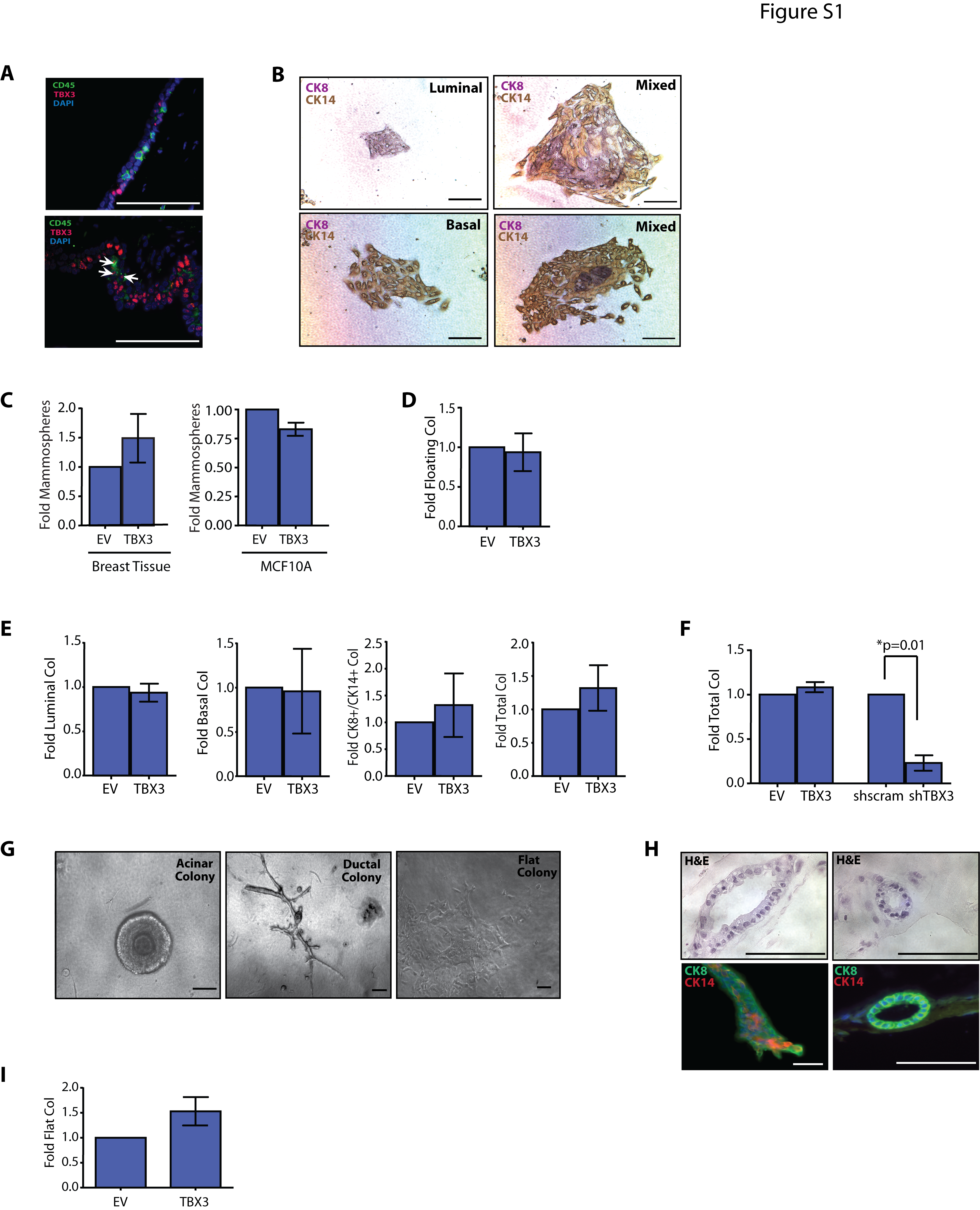

Supplement: Figure S1 — TBX3 expression does not co-localize with CD45 expressing immune cells. (A) TBX3 (red) was expressed by mammary epithelial cells as well as infiltrating CD45+ (green) immune cells in breast epithelium (arrows). Expression was detected by immunofluorescence on paraffin-embedded breast epithelial sections (n = 5 patient samples). Nuclei were counterstained with DAPI (blue). (B) Primary mammary epithelial cells (MEC) from reduction mammoplasty tissue plated at limiting dilution on adherent plates formed cytokeratin (CK) 8+ luminal colonies, CK14+ basal colonies, and mixed CK8/14+ colonies. (C) TBX3 overexpression in primary MEC (n = 3 patient samples; mean±s.e.m.) and MCF10A cells (n = 3 experiments) did not enhance mammosphere formation. (D) Overexpression of TBX3 in primary MEC did not enhance floating colony formation above adherent plates (n = 3 patient samples; mean±s.e.m.). (E) Overexpression of TBX3 in primary MEC did not enhance colony formation of any lineage on adherent plates (n = 3 patient samples; mean±s.e.m.). (F) Overexpression of TBX3 did not enhance total colony formation in MCF10A cells, however reduction in TBX3 expression resulted in significantly decreased total colony formation. Colonies were stained with crystal violet, and absorbance was quantified for 3 experiments in triplicate (mean±s.e.m.). (G) Growth of primary MEC and MCF10A cells on a collagen substrate results in the formation of 3 distinct colonies: luminal acinar, basal ductal, and flat colonies. (H) Acinar and ductal colonies growing on 3D collagen gels form a hollow lumen surrounded by a single layer of epithelial cells that demonstrate variable expression of CK8 and CK14. (I) TBX3 overexpression did not alter flat colony growth in MEC transduced with TBX3 lentivirus compared to MEC transduced with empty vector (EV; n = 8 patient samples; mean±s.e.m.). Scale bars = 100 µm. (TIF) [file pone.0111442.s001.tif]

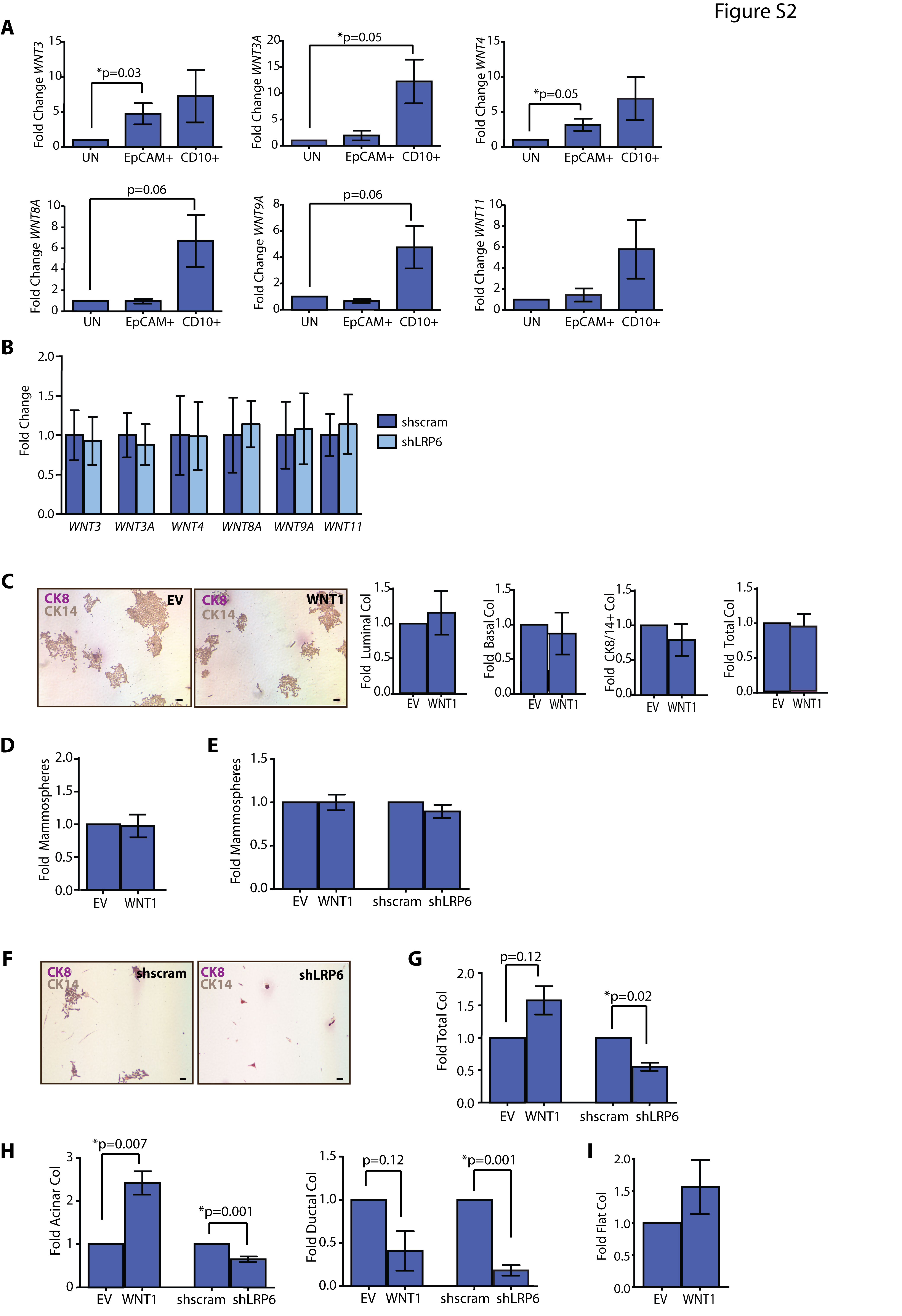

Supplement: Figure S2 — WNT1 expression increases luminal acinar progenitor cells. (A) EpCAM+ luminal cells expressed significant levels of WNT3 and WNT4, while CD10+ basal epithelial cells expressed increased levels of all WNT family ligands examined (n = 6 patient samples; mean±s.d.). Mammary epithelial cells (MECs) from reduction mammary samples were sorted and differences were detected using qPCR. (B) WNT family ligand expression was not significantly altered in MCF10A cells transduced with shLRP6 compared with control cells (n = 3 experiments; mean±s.d.). Differences detected by qPCR. (C) WNT1 expression did not alter colony formation on adherent plates in MEC infected with WNT1 or empty vector (EV) lentivirus (n = 3 patient samples; mean±s.e.m.). (D) No difference in mammosphere formation was detected between control and WNT1 infected primary MECs plated at clonal density on non-adherent plates for 7 days. (E) No differences were detected for mammosphere formation in MCF10A cells expressing WNT1 compared with EV control cells or in shLRP6 cells compared with shscrambled (shscram) control cells (n = 3 experiments; mean±s.e.m.). (F) Diminished LRP6 expression significantly decreased colony formation on adherent plates in MECs infected with shLRP6 or shscram lentivirus (n = 3 patient samples). (G) Decreased expression of LRP6 significantly decreased colony formation in MCF10A cells. Colonies were stained with crystal violet, and absorbance was quantified for 3 experiments in triplicate (mean±s.e.m.). (H) WNT1 expression in MCF10A cells significantly increased acinar colonies compared with EV control cells. Decreased LRP6 expression significantly decreased both acinar colonies and ductal colonies compared with shscram control cells (n = 3 experiments; mean±s.e.m.). (I) WNT1 expression did not significantly alter flat colony formation compared to EV controls in lentivirally transduced MEC (n = 6 patient samples; mean±s.e.m.). Scale bars = 100 µm. (TIF) [file pone.0111442.s002.tif]

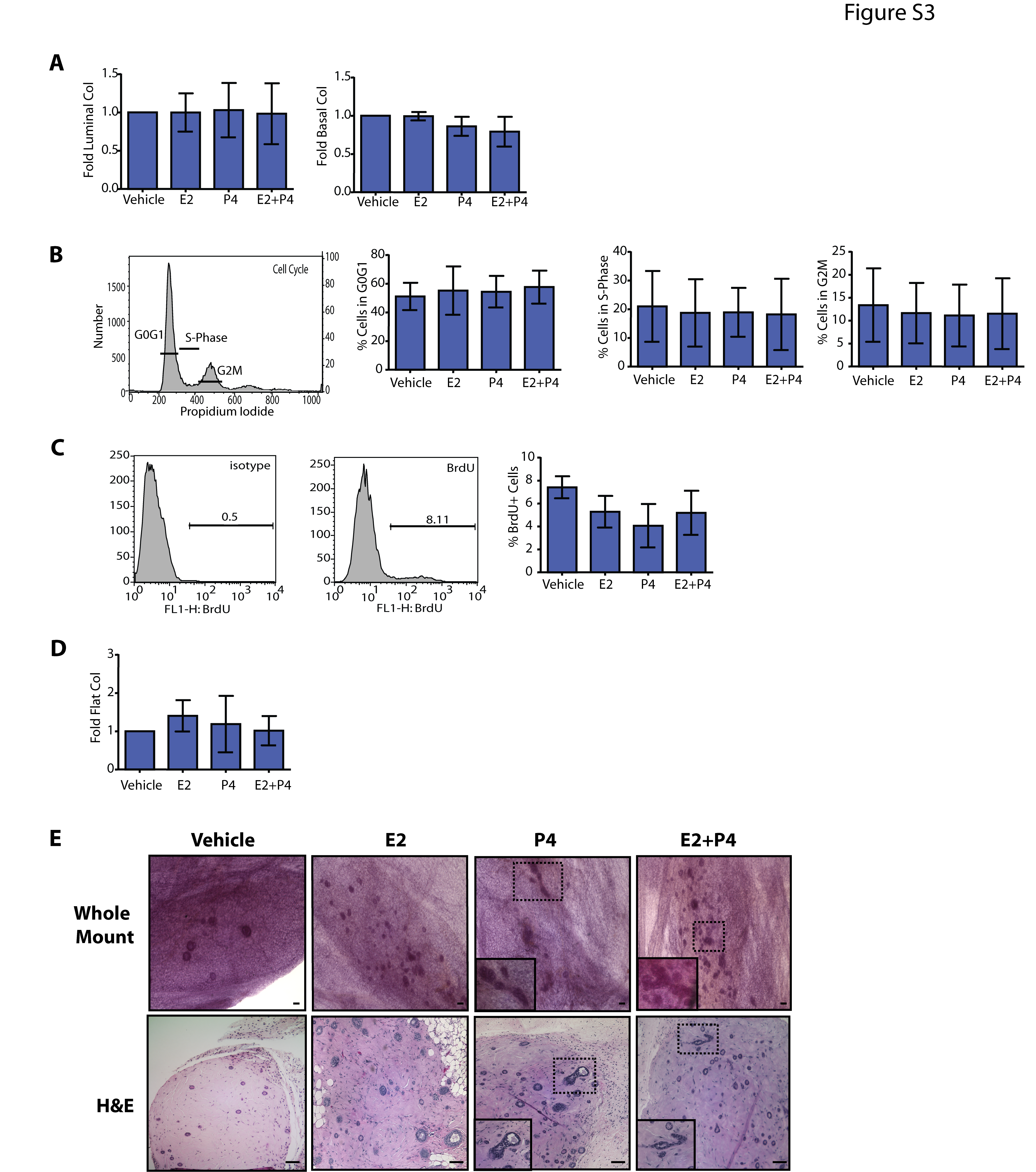

Supplement: Figure S3 — Progesterone increases the formation of ductal outgrowths in humanized mammary fat pads, related to Figure 3 . (A) Treatment of mammary epithelial cells (MECs) with 17β-estradiol (E2) and/or progesterone (P4) did not increase the number of luminal or basal colonies in adherent culture compared to those treated with vehicle (n = 3 patient samples; mean±s.e.m.). (B, C) Growth of MEC with E2 and/or P4 in adherent culture did not alter the proliferation of colonies compared with those grown with vehicle (n = 3 patient samples; mean±s.e.m.). Proliferation was assessed by flow cytometry measuring cellular populations in each portion of the cell cycle when stained with propidium iodide (B) as well as by 5-bromo-deoxyuridine (BrdU) incorporation (C). (D) MEC treated with E2 and/or P4 did not increase flat colony formation compared with those treated with vehicle (n = 9 patient samples; mean±s.e.m.). (E) Representative whole mounts and hematoxylin and eosin (H&E) stained sections from human-in-mouse (HIM) NOD/SCID mice. Primary epithelial cells (MEC) were isolated from reduction mammoplasty tissues, transduced with GFP lentivirus, and grown in the humanized fat pads of ovariectomized NOD/SCID mice treated with E2, P4, E2+P4, or placebo pellets. E2+P4 significantly enhanced the formation of acinar and ductal structures within the humanized glands. Glands from P4 and E2+P4 treated mice demonstrated increased growth of ductal structures (inset; n = 3 experiments). Scale bars = 100 µm. (TIF) [file pone.0111442.s003.tif]

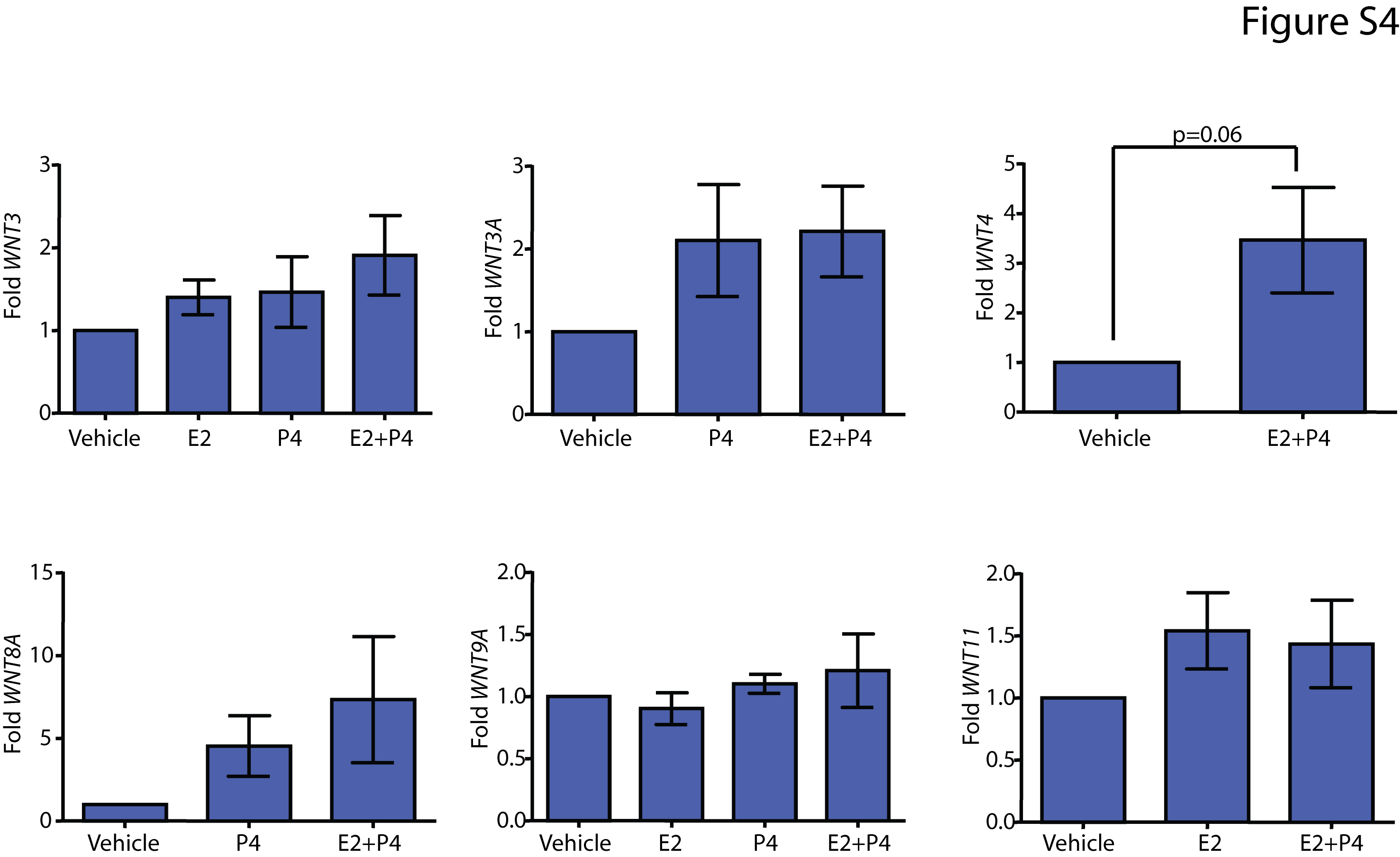

Supplement: Figure S4 — Expression of WNT family ligands in mammospheres is increased by estrogen and progesterone, related to Figure 4 . Expression of WNT family ligands was increased by treatment with 17β-estradiol (E2) and/or progesterone (P4) in primary epithelial cells grown as mammospheres compared with those treated with vehicle (n = 6 patient samples; mean±s.d.). Differences were detected by qPCR. (TIF) [file pone.0111442.s004.tif]
