# Supplementary material for: Human Breast Progenitor Cell Numbers Are Regulated by WNT and TBX3
Source: PLoS One. 2014 Oct 28;9(10):e111442. doi: 10.1371/journal.pone.0111442 (PMC4211891; doi:10.1371/journal.pone.0111442)
Supplement: Table S1 — Primers for qPCR analysis. (DOCX) [file pone.0111442.s005.docx]

**Table S1. Primers for qPCR analysis.**

|  | Forward 5’🡪3’ | Reverse 5’🡪3’ |
| --- | --- | --- |
| Estrogen Receptor Alpha | ATTGAAGTGGGCATGAGAACAT | CAATACCAACATCAGCCAGAAA |
| GAPDH | GAGTCAACGGATTTGGTCGT | TTGATTTTGGAGGGATCTCG |
| LRP5 | ATCTCCCTCGAGACCAATAACA | GCTGACGTCTGTGCCAGTAGATG |
| LRP6 | CCCATGCCCCTGGTTCTACT | CCAAGCCACAGGGATACAGT |
| Progesterone Receptor | TGGATGAGCTTAATGGTGTTTG | ATTCTTTCATCCGCTGTTCATT |
| TBX3 | GCCCAGGCTTCTTCTCCAG | GCCCAGGCTTCTTCTCCAG |
| WNT1 | TAGCCTCCTCCACGAACCTG | CAGCCTCGGTTGACGATCTTG |
| WNT2B | ATTTCCCGCTCTGGAGATTT | AAGCTGGTGCAAAGGAAAGA |
| WNT3 | AGGGCACCTCCACCATTTG | GACACTAACACGCCGAAGTCA |
| WNT3A | GCATCAAGATTGGCATCCAGG | CCCTGGTAGCTTTGTCCAGC |
| WNT4 | ACAGCTGGAAGGCTGACAGT | AGGTTGCTGGTGCTGTCTCT |
| WNT8A | GCTCTTCAGCTCTCCACCCAC | CAGCGTTCCCAAGCAAACT |
| WNT9A | TGAGAAGAACTGCGAGAGCA | CTCCACATAGCAGCACCAAC |
| WNT11 | TATCCGGCCTGTGAAGGACT | GTTGCACTGCCTGTCTTGTG |
