# Supplementary material for: Human Breast Progenitor Cell Numbers Are Regulated by WNT and TBX3
Source: PLoS One. 2014 Oct 28;9(10):e111442. doi: 10.1371/journal.pone.0111442 (PMC4211891; doi:10.1371/journal.pone.0111442)
Supplement: Table S2 — Growth in suspension enhances structural progenitor activity. (DOCX) [file pone.0111442.s006.docx]

**Table S2.** Growth in suspension enhances structural progenitor activity.

| **Structural** **Progenitors** | **Progenitor Lineage** | **Frequency** | **Fold Progenitor Enrichment^1^** |
| --- | --- | --- | --- |
| ***Following Dissociation*** | | | |
| Acinar Progenitor (N=13) | Luminal | 0.002±0.003 2/1000 |  |
| Ductal Progenitor (N=13) | Basal | 0.0009±0.0004 0.9/1000 |  |
| ***Following Non-adherent Growth*** | | | |
| From Mammospheres (N=12) | Luminal & Basal | 0.35±0.07 350/1000 | 120.7 |
| From Floating Colonies (N=12) | Luminal | 0.31±0.07 310/1000 | 155.0 |

^1^Frequency of colony growth following non-adherent growth/frequency of single cell outgrowth of colonies of the same lineage.
